# Impact of fluorination on interface energetics and growth of pentacene on Ag(111)

**DOI:** 10.3762/bjnano.11.120

**Published:** 2020-09-08

**Authors:** Qi Wang, Meng-Ting Chen, Antoni Franco-Cañellas, Bin Shen, Thomas Geiger, Holger F. Bettinger, Frank Schreiber, Ingo Salzmann, Alexander Gerlach, Steffen Duhm

**Affiliations:** 1 Institut für Angewandte Physik, Universität Tübingen, Auf der Morgenstelle 10, 72076 Tübingen, Germany; 2 Institute of Functional Nano & Soft Materials (FUNSOM), Jiangsu Key Laboratory for Carbon-Based Functional Materials & Devices and Joint International Research Laboratory of Carbon-Based Functional Materials and Devices, Soochow University, Suzhou 215123, People’s Republic of China; 3 Institut für Organische Chemie, Universität Tübingen, Auf der Morgenstelle 18, 72076 Tübingen, Germany; 4 Department of Physics, Department of Chemistry & Biochemistry, Concordia University, 7141 Sherbrooke St. West, Montreal, Quebec H4B 1R6, Canada

**Keywords:** decoupling, fluorination, metal–organic interfaces, organic pi-conjugated molecules, X-ray standing wave technique

## Abstract

We studied the structural and electronic properties of 2,3,9,10-tetrafluoropentacene (F4PEN) on Ag(111) via X-ray standing waves (XSW), low-energy electron diffraction (LEED) as well as ultraviolet and X-ray photoelectron spectroscopy (UPS and XPS). XSW revealed that the adsorption distances of F4PEN in (sub)monolayers on Ag(111) were 3.00 Å for carbon atoms and 3.05 Å for fluorine atoms. The F4PEN monolayer was essentially lying on Ag(111), and multilayers adopted π-stacking. Our study shed light not only on the F4PEN–Ag(111) interface but also on the fundamental adsorption behavior of fluorinated pentacene derivatives on metals in the context of interface energetics and growth mode.

## Introduction

The performance of organic (opto)electronic devices is strongly affected by the energy level alignment at the various interfaces in such devices [1–3]. Fluorination is a viable way to change the ionization energies (IEs) of organic semiconductor thin films [4–6], which are an important parameter for energy level alignment [7–8]. Moreover, at organic–metal interfaces, fluorination is believed to decrease the coupling strength between the substrate and the adsorbate [9–11]. However, at such interfaces, vertical adsorption heights [12–13], interface dipoles (vacuum level shifts) [9,14] and consequently the energy level alignment [15–17] are affected by fluorination. Furthermore, fluorination can change the molecular multilayer growth [18–21], which in turn affects the IEs. Overall, predicting the impact of fluorination on the energetics at organic–metal interfaces still remains a challenge.

In this context, pentacene (PEN) and perfluorinated pentacene (PFP) are frequently-studied model compounds [9,18,22–35]. PEN and PFP have almost identical optical gaps in thin films (1.85 eV and 1.75 eV, respectively) [36–37], and the experimental gas phase IEs (measured by UPS) are 6.59 eV [38] and 7.50 eV [39], respectively. This trend of the IEs is also found for thin films comprised of PEN or PFP with a flat-lying (long and short molecular axes parallel to the substrate) orientation, which have IEs (in monolayers on graphite) of 5.65 eV and 6.20 eV, respectively [25–26]. The decrease in the IE is due to solid state polarization, which is a general phenomenon for molecular thin films [40–44]. However, in thin films comprised of molecules in a standing orientation on SiO_2_ (long molecular axis perpendicular to the substrate), the IE of PEN decreases to 4.90 eV and the IE of PFP increases to 6.65 eV [45]. The opposite trend of the orientation dependency of the IEs has its origin in the strongly polar C–F bonds of PFP [5,27], which reverse the intramolecular quadrupole moment of PEN and PFP [46]. The collective influence of these quadrupoles in turn affects the potential energy, and thus the IEs of the thin films, an effect that is likewise termed electrostatic contribution polarization [11,44].

Perfluorination does not impact the orientation of PEN and PFP in the contact layer with clean metals, where both compounds are lying flat [9,30–31,47–52]. On Au(111), the coupling strength of both monolayers with the substrate is rather similar and physisorptive [29,53]. On Cu(111), PFP shows a behavior close to physisorption [9], although the coupling strength might be slightly stronger than with Au(111) [54]. PEN on Cu(111), however, is strongly chemisorbed, involving a partial filling of the former lowest unoccupied molecular orbital (LUMO) by a charge transfer from the substrate [9,28]. This can be explained by the repulsive interaction of the fluorine atoms with the substrate, which leads to much larger vertical adsorption heights of PFP compared to PEN in monolayers on Cu(111) [9]. Ag(111) represents an intermediate case [55] with weak chemisorption of PEN [28,51] and physisorption of PFP [50]. Multilayers of PEN adopt a herringbone arrangement on virtually all substrates [47,56–59], whereas PFP multilayers exhibit π-stacking [18,56]. The different multilayer packing motifs of PEN and PFP can again be explained by electrostatic intramolecular interactions [18].

Overall, the influence of perfluorination on the multilayer growth and on the interfacial coupling of pentacene on clean metal surfaces is understood to a large extent. However, the impact of partial fluorination is less well studied, and thus we explored thin films of partially fluorinated PEN, namely 2,3,9,10-tetrafluoropentacene (F4PEN) [46,60–61]. F4PEN physisorbs on Au(111) [62] and chemisorbs on Cu(111), involving interfacial charge transfer and strong molecular distortions [63]. Here, we investigated the coupling with Ag(111) as we expected this to be an interesting intermediate case. We determined the vertical adsorption heights of F4PEN (sub)monolayers on Ag(111) employing the XSW technique [64–67]. The lateral order in the monolayer was determined by LEED. Possible chemical interactions between F4PEN and the substrate were studied by XPS. The energy level alignment was investigated by UPS. Furthermore, we compared the adsorption behavior of F4PEN on Ag(111) with that of PEN and PFP on the same substrate to understand the influence of fluorine substitution on the interfacial electronic structure of prototypical pentacene derivatives at organic–metal interfaces.

## Results

**Figure 1:**
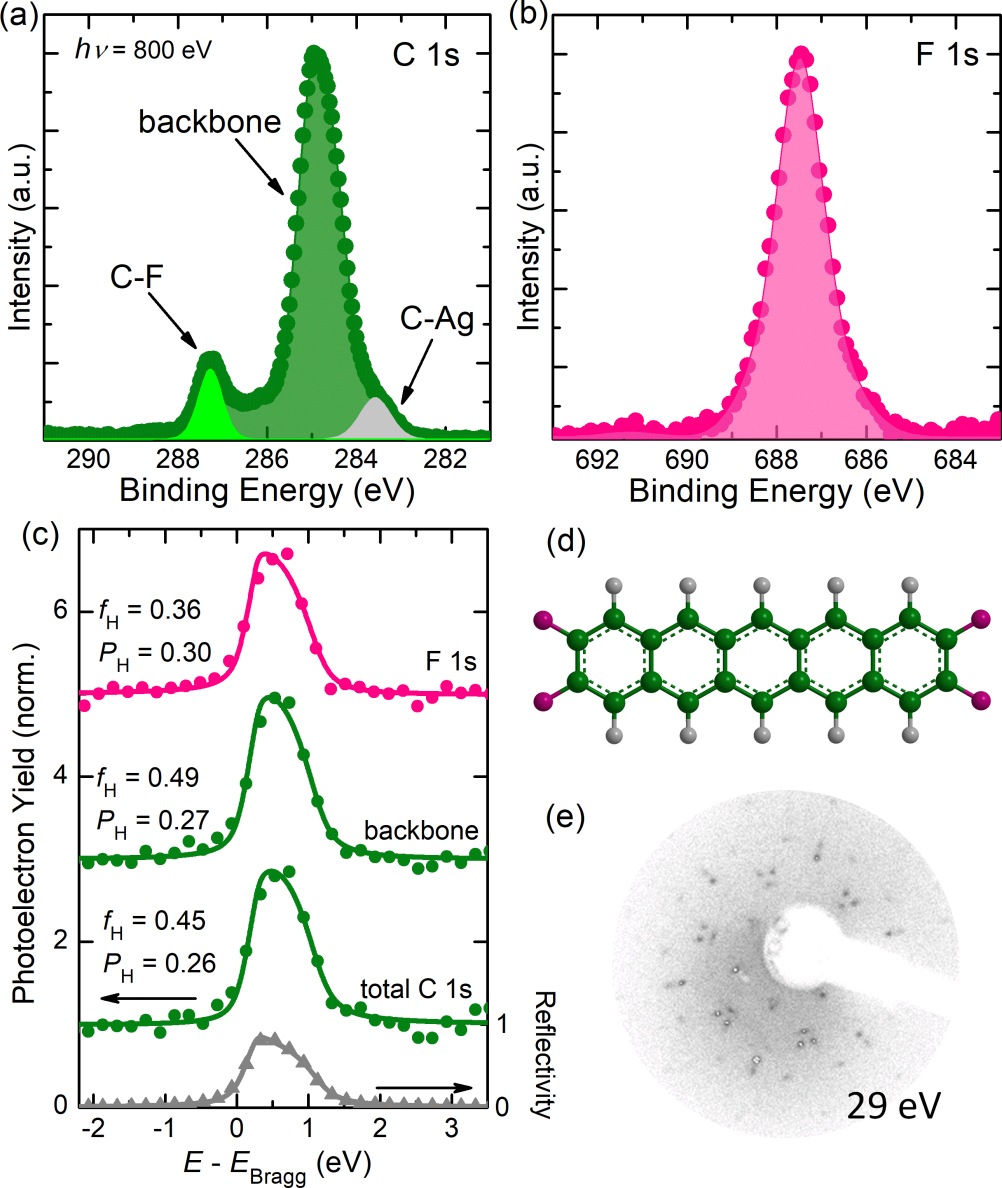
(a) C 1s and (b) F 1s core levels of F4PEN in monolayers on Ag(111) measured at DLS. (c) Reflectivity and photoelectron yield (*Y*_p_) as a function of the photon energy (*h*υ) relative to the Bragg energy (*E*_Bragg_ = 2630 eV) for a (sub)monolayer (<2 Å) F4PEN thin film on Ag(111). For each element, the coherent position (*P*_H_) and the coherent fraction (*f*_H_) are given. (d) Chemical structure of F4PEN. (e) LEED pattern for F4PEN on Ag(111) with a nominal thickness of 4 Å, measured at 295 K with a beam energy of 29 eV. The LEED pattern is almost the same as for PEN on Cu(111) [28].

The determination of the vertical adsorption heights of F4PEN in (sub)monolayers on Ag(111) relied on high-resolution core level spectra, which are shown in Figure 1 (additional XPS spectra are shown in Supporting Information File 1, Figure S1). Following the assignment of the F4PEN core levels on Cu(111) [63], the C 1s peak centered at 287.29 eV binding energy (BE) was assigned to the carbon atoms bound to the fluorine atoms (C–F), and the main peak centered at 284.88 eV BE was assigned to the carbon atoms in the backbone of F4PEN (C–C). In addition, at the low-BE edge a small tail attributed to the carbon atoms bound to the substrate (C–Ag) belonged to the broken C–F bonds due to the dehalogenation reaction [63,68]. The symmetric F 1s peak was centered at 687.47 eV BE. Figure 1 shows the photoelectron yield (*Y*_p_), i.e., the photoemission intensity of the core-levels as a function of the photon energy, which allowed to determine the coherent position (*P*_H_) and the coherent fraction (*f*_H_) of the adsorbate atoms [66,69]. The former gave the adsorption distance in terms of the lattice spacing of the silver substrate: *d*_H_ = *d*_0_(*n* + *P*_H_) (typical precision < 0.05 Å), with *n* being an integer. The coherent fraction 0 ≤ *f*_H_ ≤ 1 describes the degree of vertical order of the adsorbate atoms, with *f*_H_ = 0 for a completely disordered system and *f*_H_ = 1 for all probed adsorbate atoms having the same adsorption distance. XSW measurements were performed for two (sub)monolayer coverages of F4PEN on Ag(111), yielding essentially the same results (the data for higher coverage is shown in Supporting Information File 1, Figure S2). F4PEN adsorbed in an essentially planar geometry, with averaged vertical adsorption distances of around 3.00 Å for carbon and fluorine atoms. The adsorption distances are summarized in Table 1, together with literature values for PEN and PFP. In general, the adsorption distance of F4PEN in (sub)monolayers on Ag(111) was similar to that of PEN and PFP on the same substrate.

**Table 1:** Summary of the element-specific vertical adsorption heights (*d*_H_/Å) of (fluorinated) pentacene in (sub)monolayers on Ag(111) measured with the XSW technique. A low coverage indicates the submonolayer (<2 Å) and a high coverage indicates an almost closed monolayer (≈3 Å). The values for PEN and PFP are taken from [51] and [50], respectively.

coverage	element	PEN	F4PEN			PFP
		
		*d*_H_	*f*_H_	*P*_H_	*d*_H_	*d*_H_

low	C	2.98	0.49	0.27	3.00 ± 0.03	3.16
	F	–	0.36	0.30	3.05 ± 0.02	3.16
high	C	3.12	0.37	0.26	2.97 ± 0.02	–
	F	–	0.45	0.24	2.93 ± 0.01	–

Turning from vertical to lateral order, the LEED pattern of F4PEN on Ag(111) is shown in Figure 1e. At a nominal thickness of 4 Å, F4PEN is well-ordered, in contrast to PEN on the same substrate, which is disordered at room temperature for the same thickness [28,31,52]. Interestingly, the LEED pattern of F4PEN on Ag(111) was virtually identical to those of PEN on Cu(111) [28]. Increasing the coverage to nominally 48 Å did not significantly change the LEED image (see Supporting Information File 1, Figure S3). This indicated that increasing the coverage does not change the lateral order at the contact layer and pointed towards Stranski–Krastanov growth (island on wetting layer) [70] since the signal from the interface was visible even when a nominal coverages corresponding to several layers was deposited. Stranski–Krastanov growth has been suggested for F4PEN on Cu(111) [63], and furthermore was supported by thickness-dependent XPS, where the relative intensity barely changed as the nominal F4PEN thickness increased from 8 to 48 Å (for the spectra see Supporting Information File 1, Figure S4). Furthermore, a rigid shift of the C 1s and F 1s core levels of around 0.2 eV to a higher BE could be observed for a nominal thickness from 2 to 48 Å. This shift could be attributed to the screening effect (also often called the mirror force effect), which is commonly observed in photoemission data of organic thin films on metal substrates [71–73]. The absence of nonrigid shifts of the core level peaks between the mono- and multilayer coverage, which usually occur in the case of chemisorption [74–77], pointed to a weak interfacial coupling.

**Figure 2:**
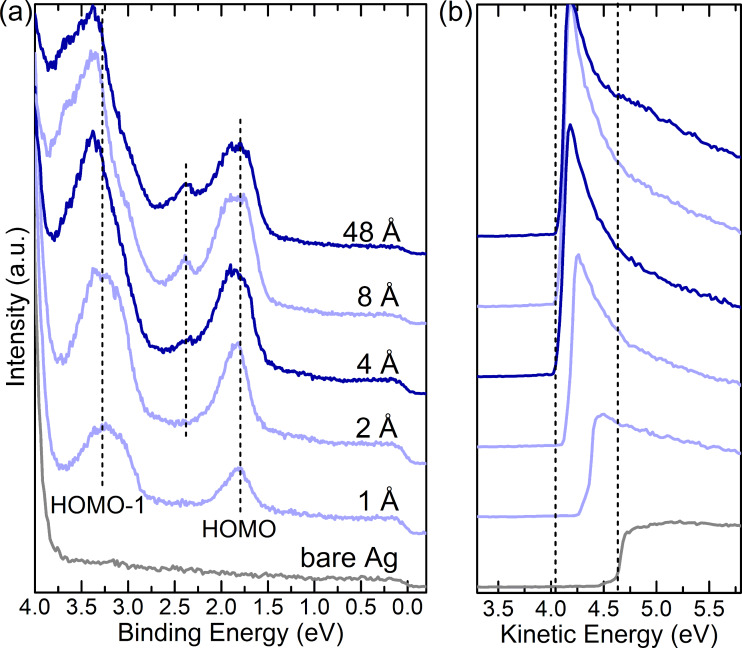
Valence band (a) and secondary electron region (b) of the UPS spectra of F4PEN on Ag(111). In (a), the nominal mass thickness is denoted.

Figure 2a shows the UPS data for the valence band region of stepwise deposited F4PEN on Ag(111). For a nominal thickness of 1 Å, two peaks that could be assigned to the HOMO and HOMO−1 levels of F4PEN were visible. The low BE onset of the HOMO-derived peak was located at a 1.56 eV BE. For a thicknesses of 4 Å and larger, a third peak emerged at the high BE shoulder of the HOMO-derived peak. Interestingly, the maxima of these two peaks did not change with an increasing coverage (as highlighted by the vertical lines in Figure 2a). However, the HOMO-derived peak broadened, and for a nominal thickness of 48 Å, its onset was at 1.51 eV BE. The HOMO−1-derived peak also broadened, but its maximum showed a shift to higher BE with increasing coverage. This shift was essentially parallel to that of a deeper-lying valence electron feature (centered at around a 10 eV BE, the spectra are shown in Supporting Information File 1, Figure S5) and to that of the core levels. Possible reasons why the HOMO level did not show the screening effect and the origins of the third peak will be discussed below.

The secondary electron cut-off (SECO) spectra (Figure 2b) allowed to determine the VL position above the Fermi level (*E*_F_), which was reduced from an initial value of 4.62 eV [i.e., the work function of clean Ag(111)] to 4.05 eV for a nominal F4PEN thickness of 4 Å and stayed essentially constant when increasing the coverage. The VL decrease of 0.57 eV was rather similar to that of PEN or PFP thin films on the same substrate [28,50] and could be mainly attributed to the so-called push-back effect, i.e., the reduction of the surface dipole part of the metal work function [78] by the mere presence of the molecular adsorbate [73,79–80]. The VL and the HOMO onset gave the IE, which decreased from 5.87 eV for the monolayer (4 Å) to 5.56 eV for the multilayer (48 Å).

## Discussion

**Figure 3:**
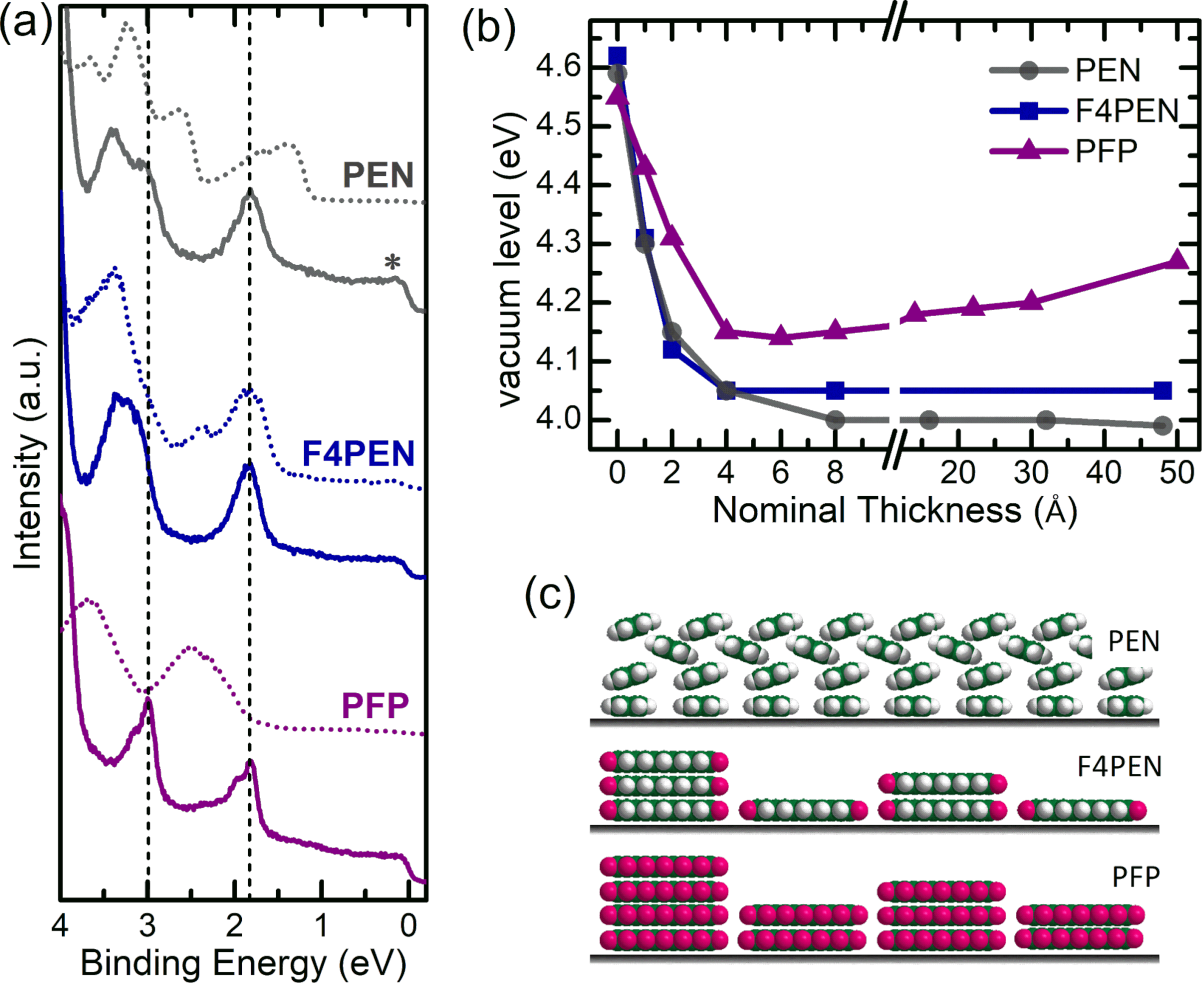
(a) Valence band spectra for (fluorinated) pentacene in (sub)monolayer (solid lines) and multilayer (dotted lines) thin films on Ag(111). (b) Evolution of the vacuum level of (fluorinated) pentacene with increasing thickness on Ag(111). The PEN and PFP data in (a) and (b) is taken from [28,50]. (c) Hypothetical growth modes of (fluorinated) pentacene on Ag(111) on profile view, with the C atoms in green, the F atoms in red and the H atoms in grey. Note, that PEN (F4PEN and PFP) is viewed along the short (long) molecular axis. A more detailed discussion on the F4PEN adsorption geometry on Ag(111) based on the analysis of the *f*_H_ values can be found in Supporting Information File 1, Figure S6.

Figure 3 summarizes the experimental results of F4PEN thin films on Ag(111) and compares them with those of PEN and PFP thin films on the same substrate. Notably, the valence band spectra were almost identical for the monolayer coverages. In all cases, the HOMO-derived peak was centered at 1.85 eV, while the peak centered at a higher BE (≈3.00 eV) was identified as the HOMO−1-derived peak. In addition, the vertical adsorption geometries were rather similar (Table 1), and no obvious distortions became evident for the fluorinated compounds, which drew the picture of lying molecules (PEN, F4PEN, and PFP) in monolayers on Ag(111). The carbon atoms of PFP [50] exhibited a slightly larger adsorption distance compared to PEN (low coverage) [51] and F4PEN. The increase in *d*_H_ for PEN by raising the coverage was explained by a vertical ordering due to intermolecular interactions [51]. The small coverage-dependent changes of F4PEN showed an opposite trend, but the change in the vertical adsorption heights of the carbon atoms was only 0.02 Å, i.e., within the experimental uncertainty. In particular, the fluorine atoms showed a difference in the adsorption distance of Δ*d*_H_ = 0.10 Å. For the higher coverage (see Supporting Information File 1, Figure S2), the fluorine atoms were apparently located below the carbon atoms. However, such a behavior would be rather unusual as for F4PEN as well as for PFP on Cu(111), an upward bending of the fluorine atoms was observed [9,63] and is also expected due to the repulsive interaction of the fluorine atoms with the metal surface [81]. Moreover, also for related peripherally fluorinated molecules on the (111) surfaces of coinage metals, a downward bending of the fluorine atoms has never been observed [64]. In particular, this holds for PFP on Ag(111) [50] and F_16_CuPc on the same substrate [82]. Taking into account the error bar of our measurements, a lying adsorption geometry will be considered in the following. For a higher coverage, F4PEN adsorbed at a lower position than PEN, which seemed to be at odds with the expected decrease of the organic–metal coupling strength by fluorination. However, also the carbon atoms of F4PEN on Cu(111) have smaller vertical adsorption distances (2.24 Å [63]) than PEN (2.34 Å [9]). The adsorption distance of the fluorine atoms of F4PEN on Cu(111), however, was 3.40 Å, and thus even higher than that of the PFP fluorine atoms on the same substrate (3.08 Å [9]).

The similar adsorption distances on Ag(111) and the almost planar adsorption geometry could explain the similar vacuum level shifts (around 0.5 eV) upon monolayer formation of the three molecules (Figure 3): In the absence of notable organic–metal interactions, the interface dipole is mainly due to the pushback effect, and its magnitude is determined by the adsorption distance [83–84]. In all cases, the vacuum level shift saturated at a nominal thickness of 4 Å. This thickness thus represented an almost closed monolayer coverage, and subsequently deposited molecules grew predominantly in multilayers (and not on possibly uncovered substrate patches).

The almost identical HOMO positions were unexpected and in contrast to the monolayers of PEN and PFP on graphite, with a likewise lying-down orientation and large differences in the HOMO positions [11,25–26]. In general, for a strong interfacial coupling and charge transfer, the resonance structure of the adsorbate in the monolayer can be notably different from that in multilayers [75,85–87]. For the specific case of the pentacene derivatives on Ag(111), strong coupling was not observed. However, the interaction of PEN with Ag(111) was termed “soft chemisorption” [51], and by a closer look at the PEN monolayer spectrum in Figure 3a, a faint peak close to *E*_F_ (marked with an asterisk) could be ascribed to a charge transfer from the substrate [28]. The transport gap of PEN is 2.20 eV [88]. Similar transport gaps can be expected for PFP and F4PEN, which puts the Fermi level rather close to the LUMO. Moreover, in the vicinity of a metal surface, the gap has been found to decrease, and the molecular energy levels become broadened [1,89–90], which is expected to promote the charge transfer to the LUMO [7–8].

The positions of the HOMO levels of molecular monolayers on metal substrates depend also on the magnitude of the above mentioned screening effect [10,72,89] or, more generally, on the electronic polarization of the charged molecules on the surfaces. In addition to the substrate contribution, interactions with neighboring molecules, which have an electrostatic and an induction contribution, have to be taken into account [11,44]. Induction interactions are always stabilizing the photohole and should–given the same coverage–lead to similar shifts for PEN, F4PEN and PFP. As mentioned in the introduction, the electrostatic contribution depends on intramolecular quadrupole moments and has, due to the strong polar character of the C–F bonds, opposite signs for PEN and PFP [11]. Electrostatic potential maps of PEN, F4PEN and PFP in the gas phase can be found in [46]. In general, hydrogen atoms bear a minimum, and fluorine atoms a maximum of the electron density, i.e., the quadrupole moments of F4PEN along the molecular short and long axes have opposite signs, while they have the same signs for PEN and PFP, respectively. Without precise knowledge of the F4PEN monolayer structure, the electronic polarization cannot be calculated, but it is reasonable to assume that the interplay of substrate, induction and electrostatic contributions resulted in the observed identical HOMO level position in all three monolayers.

For an in-depth discussion of the multilayer electronic structure, for which the valence electron spectra were strikingly different (Figure 3a), knowledge about the morphology and the molecular orientation was indispensable [91–94]. As mentioned above, multilayers of PEN show a herringbone arrangement on virtually all substrates [47,56–59], and for commonly prepared vacuum-sublimed thin films on metals, the long molecular axis is parallel to the substrate [95–97]. Indeed, for the PEN multilayers on Ag(111), the HOMO-derived peak measured by partly angle-averaging UPS exhibited the typical shape of crystalline PEN in a herringbone arrangement [28,98–100]. The shift of the HOMO onset to lower BE could be explained by the reduced IE of PEN thin films consisting of tilted instead of lying molecules [28–29]. PFP, in contrast, exhibits π-stacking on various substrates [18,56]. The X-ray scattering data shown in Supporting Information File 1, Figure S7 confirmed this ordering motif for the Ag(111) surface. The almost symmetric shape of the PFP HOMO in multilayers on Ag(111) resembled that of likewise π-stacked organic multilayer thin films [101–103]. The shift to higher BE could, in part, be attributed to the screening effect [50], and the broadening could be ascribed to the intermolecular band dispersion [18]. The multilayer structure of F4PEN on Ag(111) is unknown, but the comparison of photoelectron spectroscopy data with that of PEN and PFP hinted at π-stacking: For F4PEN, a herringbone arrangement involving an inclination of the molecular short axis would expose the C–H bonds (and not the C–F polar bonds) to the surface. This case would be in analogy to PEN, and a shift of the HOMO towards a lower BE should be expected, which was, however, not observed. π-stacking, on the other hand, could explain the photoelectron spectroscopy data: The rigid shifts in UPS and XPS were due to screening by the substrate; the valence electron peak between the HOMO and the HOMO−1 for multilayer coverage had almost the same BE as the HOMO in PFP multilayers.

## Conclusion

In conclusion, in the monolayer regime on Ag(111), (partial) fluorination of pentacene did not notably affect the adsorption geometry and the energy level alignment. This finding was most likely due to the interplay of the substrate, induction and electrostatic contributions to the solid state polarization, i.e., a mutual compensation of different mechanisms. Our results show that the rationale of “decoupling by fluorination” required a threshold of organic–metal interaction strength, as can be seen by the monolayers of PEN, F4PEN and PFP on Cu(111), which were indeed distinctively different [9,63]. Moreover, the strong intramolecular polar C–F bond had an eminent impact on the multilayer structure of the pentacene derivatives on Ag(111), which were π-stacked for PFP and F4PEN, whereas PEN adopted a herringbone arrangement. For PFP this could be ascribed to attractive quadrupole interactions between adjacent PFP molecules [18,56], and this seemed to be the case for partial fluorination as well. The differences in thin film structure were also reflected in the electronic structures, which were distinctively different in multilayers on Ag(111). Our results highlight that even for weak organic–metal interaction, the fluorine substitution significantly affects the organic thin film growth beyond the first layer as well as the multilayer electronic structure.

## Experimental

F4PEN was synthesized following an established procedure [60] and vacuum-sublimed on clean metal surfaces (prepared by repeated Ar^+^ ion sputtering and annealing cycles [up to 550 °C]), with deposition rates of about 0.5 Å/min. The film mass thickness was monitored with a quartz crystal microbalance (QCM) near the sample, and a nominal thickness of 4 Å is close to monolayer coverage. The high-resolution XPS (HR-XPS) and normal-incidence XSW (NIXSW) experiments were performed at beamline I09 at Diamond Light Source (DLS, UK) using both the soft (110–1100 eV) and hard (2.1–18 keV) X-ray beams [104–105]. Sample preparation and measurements were performed in situ under ultrahigh vacuum (UHV) conditions. The analysis chamber (base pressure: 3 × 10^−10^ mbar) contained a VG Scienta EW4000 HAXPES hemispherical photoelectron analyzer, which was mounted at 90° relative to the incident X-ray beam. The reflectivity and photoelectron core level spectra of all elements were recorded at different photon energies *E* (31 data sets) within a ±3 eV interval around the normal-incidence Bragg energy *E*_Bragg_ (2630 eV) of Ag(111). The photoelectron yield *Y*_P_ (*E* − *E*_Bragg_) and the reflectivity were modeled taking into account the experimental geometry and the nondipole corrections associated with it [82]. For HR-XPS, the photon energy was 800 eV, and the angle between the incoming beam and the substrate normal was 60°. Thickness-dependent UPS and XPS experiments were carried out at Soochow University in an ultrahigh vacuum system consisting of three interconnected chambers (base pressure: 3 × 10^−10^ mbar) for substrate preparation, thin film evaporation and analysis. LEED was performed using a Micro-Channel-Plate LEED (OCI BDL800IR-MCP). Photoelectron spectroscopy experiments were performed using a SPECS PHOIBOS 150 analyzer and monochromatized He I radiation (21.22 eV) and the monochromatized Al Kα line (1486.6 eV) for UPS and XPS, respectively. For UPS, the energy resolution was 80 meV, and the angle between the incident beam and the sample surface was fixed to 40°. The spectra were collected at photoelectron take-off angles of 45°, with an acceptance angle of ±12°. A sketch of the measurement geometry can be found in Reference [28]. The XPS and UPS valence band spectra were plotted with respect to *E*_F_. In the plots of the secondary electron region of UPS (measured in normal emission), the energy scale was corrected by the applied bias voltage (−3 V) and the analyzer work function. Thus, the position of the SECO corresponds to the vacuum level (VL) with respect to *E*_F_. All organic thin film preparation steps and all measurements were performed at room temperature (295 K).

## Supporting Information

Comparison of low- and high-coverage F4PEN on Ag(111), both HR-XPS and XSW results, full spectra of coverage-dependent XPS results, C 1s and F 1s core levels, full UPS survey spectra, XRD measurement and analysis of PFP on Ag(111) and a set of XSW measurements.

File 1 Additional experimental data.
