# Supplementary material for: Impact of fluorination on interface energetics and growth of pentacene on Ag(111)
Source: Beilstein J Nanotechnol. 2020 Sep 8;11:1361–70. doi: 10.3762/bjnano.11.120 (PMC7492695; doi:10.3762/bjnano.11.120)
Supplement: File 1 — Additional experimental data. [file Beilstein_J_Nanotechnol-11-1361-s001.pdf]

BEILSTEIN JOURNAL OF NANOTECHNOLOGY

Supporting Information

for

Impact of fluorination on interface energetics and growth of pentacene on Ag(111)

Qi Wang, Meng-Ting Chen, Antoni Franco-Cañellas, Bin Shen, Thomas Geiger, Holger F. Bettinger, Frank Schreiber, Ingo Salzmann, Alexander Gerlach and Steffen Duhm

Beilstein J. Nanotechnol. **2020,** *11,* 1361–1370. doi:10.3762/bjnano.11.120

Additional experimental data

License and Terms: This is a supporting information file under the terms of the Creative Commons Attribution License (https://creativecommons.org/licenses/by/4.0). Please note that the reuse, redistribution and reproduction in particular requires that the authors and source are credited.
The license is subject to the *Beilstein Journal of Nanotechnology* terms and conditions: (https://www.beilstein-journals.org/bjnano)

XSW measurements:

The XSW technique, among other applications [1,2], is used to determine the adsorption distances of organic adsorbates deposited on the single crystals with chemical sensitivity and high precision [3]. Under the Bragg condition, an incoming X-ray beam interferes with its reflected beam and creates a standing wave field in the overlapping region, which extends above the crystal surface, usually high quality metal single crystals. As the standing wave field is generated under the Bragg condition of the taken single crystal, the distance in the field between node (or antinode) is the same as the crystal lattice space ($d_{XSW} = d_{hkl}$). When scanning the photon energy around the Bragg condition, the intensity of the local field shifts. Thus, the atoms (of the crystal/adsorbate) will experience a varying intensity, subsequently influencing their X-ray adsorption. As the photoelectrons are collected by the analysis in the meantime, the peak area in the XPS spectra, corresponds to the photoelectron yield, within the dipole approximation, is proportional to the X-ray absorption. Therefore, by determining the Y_P modulation for a given species and the photon energy-dependent reflectivity (R) around the Bragg condition, it is able to fit the data by:

$$Y_P(hv) = 1 + S_R R + 2\sqrt{R} f_H \cos(v - 2\pi P_H)$$

where S_R is the factor which corrects the dipolar effect, and v is the energy-dependent phase factor. S_R can be determined by

$$\tan\psi = \frac{S_R - 1}{S_R + 1}\tan\Delta$$

the XSW phase ψ is a simple and unique function of the partial phase shift Δ. The coherent fraction (f_H) and coherent position (P_H) are introduced in the main text. Finally, the adsorption distance (d_H) can be accessed by the relation:

$$d_H = d_{hkl}(n + P_H)$$

The XSW fits are processed by the Scilab [4] script provided by T.-L. Lee, with the non-dipolar corrections included. Due to the relatively large acceptance angle of the analyzer, non-dipolar correction [5,6] for an emission angle of 15° were included to the fit as a way to account for those electrons entering the analyzer away from grazing emission.

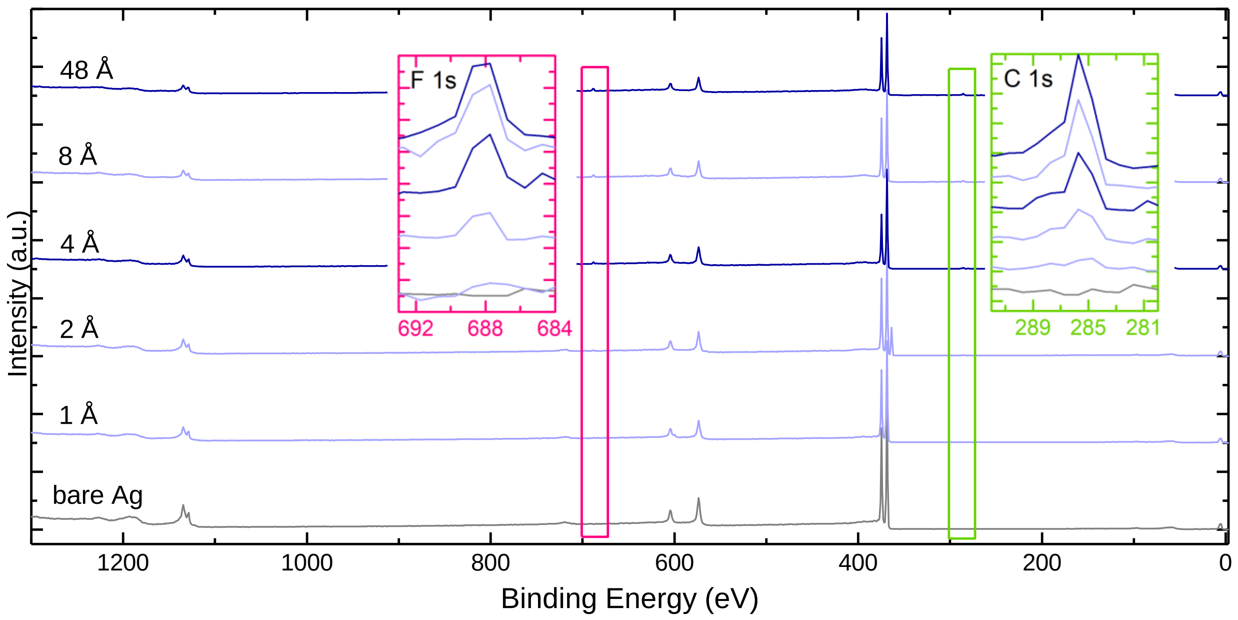

Figure S1: Thickness-dependent XPS full spectra of F4PEN on Ag(111). The nominal mono- (4 Å) and multi- (48 Å) layer curves are highlighted by darker color. The core-level spectra of C 1s and F 1s are extracted, with such a larger energy step during measurement, they are only used to define the thickness variation.

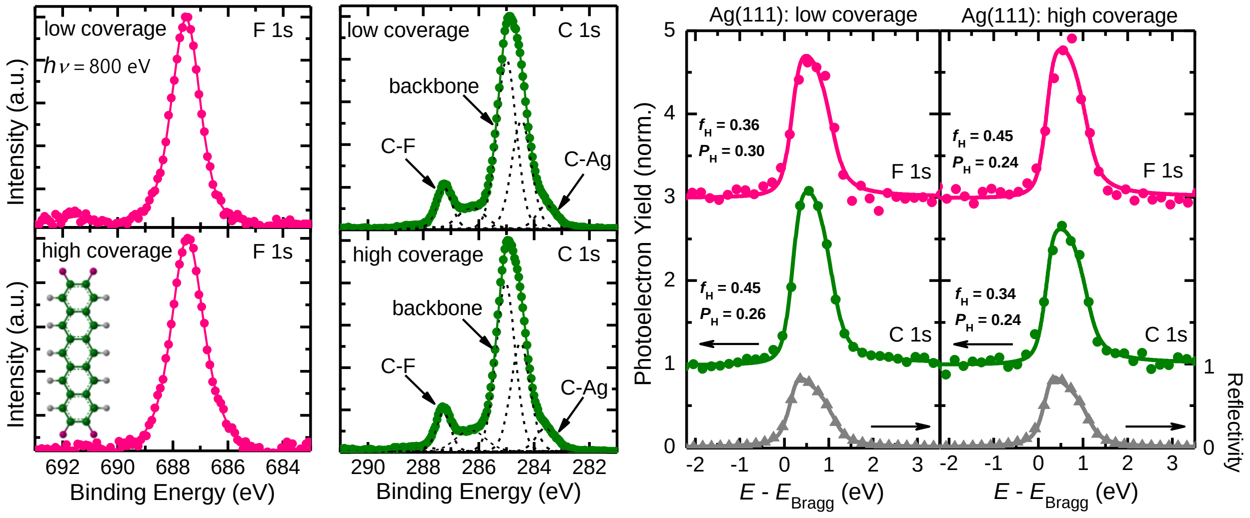

Figure S2: Left: XPS core-level spectra of F 1s (pink curve) and C 1s (green curve) with the chemical structure inserted, at low coverage and high coverage, respectively. Right: XSW photoelectron yield curves of low coverage and high coverage, "C 1s" includes the area of "C-F" and "backbone" peaks of in the XPS fitting spectra. Low coverage indicates the submonolayer (<2 Å) and high coverage indicates an almost monolayer (~3 Å).

Table S1: Summary of element-specific vertical adsorption information, including coherent fraction (f_H), coherent position (P_H) and adsorption distance (d_H/Å) of (fluorinated) pentacene in (sub)monolayers on Ag(111) measured with the XSW technique, low coverage indicates the submonolayer (<2 Å) and high coverage indicates an almost monolayer (~3 Å). Values for PEN and PFP are taken from Refs. [7] and [8], respectively.

coverage	element		PEN	F4PEN	PFP
low	C	f_H	0.65	0.49	0.25
		P_H	0.26	0.27	0.34
		d_H	2.98	3.00±0.03	3.16
	F	f_H	-	0.36	0.27
		P_H	-	0.30	0.34
		d_H	-	3.05±0.02	3.16
high	C	f_H	0.65	0.37	-
		P_H	0.32	0.26	-
		d_H	3.12	2.97±0.02	-
	F	f_H	-	0.37	-
		P_H	-	0.45	-
		d_H	-	2.93±0.01	-

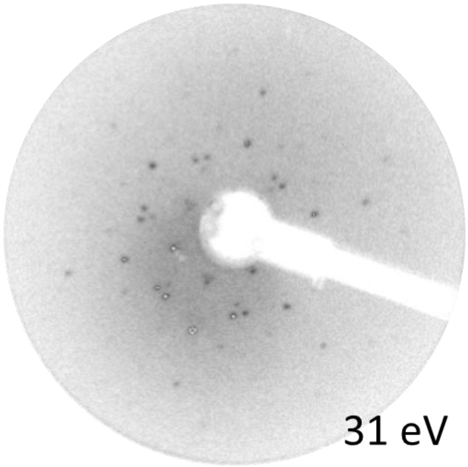

Figure S3: LEED pattern of multilayer (48 Å) coverage F4PEN on Ag(111), measured with a beam energy of 31 eV. It has the same diffraction pattern as the monolayer regime in Fig 1(e), while the diffraction spots are visible points towards Stranski–Krastanov growth.

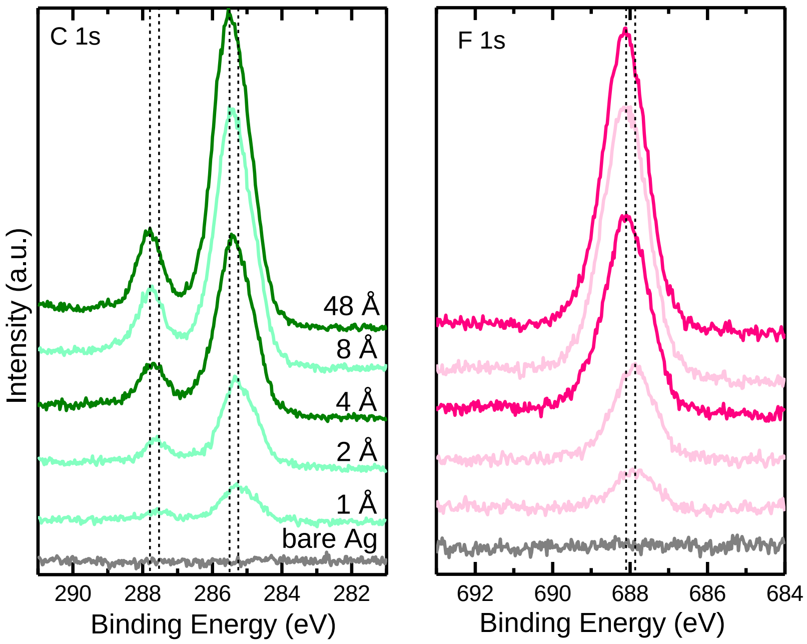

Figure S4: Thickness dependent XPS core-level spectra of F4PEN on Ag(111), C 1s (green) and F 1s (pink) spectra are displayed. The nominal mono- (4 Å) and multi- (48 Å) layer curves are highlighted by darker color. Dashed lines are added for comparison of the binding energy shift.

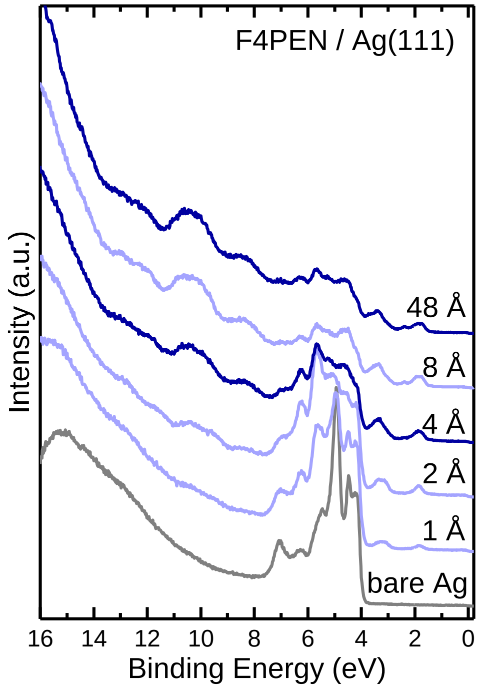

Figure S5: Thickness-dependent UPS full spectra of F4PEN on Ag(111) with increasing thickness. The nominal mono- (4 Å) and multi- (48 Å) layer curves are highlighted by darker color. With F4PEN deposition, silver signals gradually submerge and F4PEN signals appear.

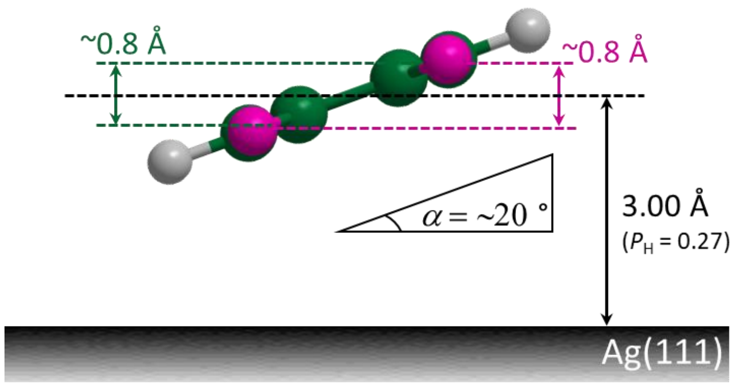

Figure S6: The sketch of F4PEN adsorbed on Ag(111) explaining the relatively low f_H values in the experiment. Our simulation suggests that the molecules are tilted less than ~20° around the long molecular axis. The schematic show a view along the long molecular axis of F4PEN (molecular size ~ 5.44*16.28 Å^2).

Table S2: Possible tilt angle based on the obtained coherent fraction (f_H), without considering the molecular dynamic and the static disorder.

coverage	element	F4PEN on Ag(111)				
		f_H	P_H	d_H	Δd_H	*tilted angle/°*
low	C	0.49	0.27	3.00±0.03	0.38	~22
	F	0.36	0.30	3.05±0.02	0.44	~19
high	C	0.37	0.26	2.97±0.02	0.45	~26
	F	0.45	0.24	2.93±0.01	0.39	~17

Generally, there are three factors which influencing the coherent fraction (f_H), (A) molecular tilt, (B) molecular dynamic and (C) the static disorder, the relation among the three factors is:

$$f_H = f_{tilt} \times f_{dynamic} \times f_{static}, (0 < f < 1)$$

In general, all three sources of reduction of the coherent fraction are expected to be present in the system, which makes a quantitative deconvolution difficult. Nevertheless, using an established procedure [9], we can estimate a maximum difference (Δd_H) of a given atom from the average adsorption distance (d_H), as summarized in the table above. Based on our simulation and the experimental coherent fraction we estimate that the tilt angle around the *long* axis of the F4PEN molecule on Ag(111) is less than 20 degree. We emphasize that this is to be considered rather as an *upper* limit for a tilt about the long axis if the reduction in coherent fraction were to be dominated by (A). Considering the other contributions ((B) and (C)), the true tilt is likely to be substantially smaller.

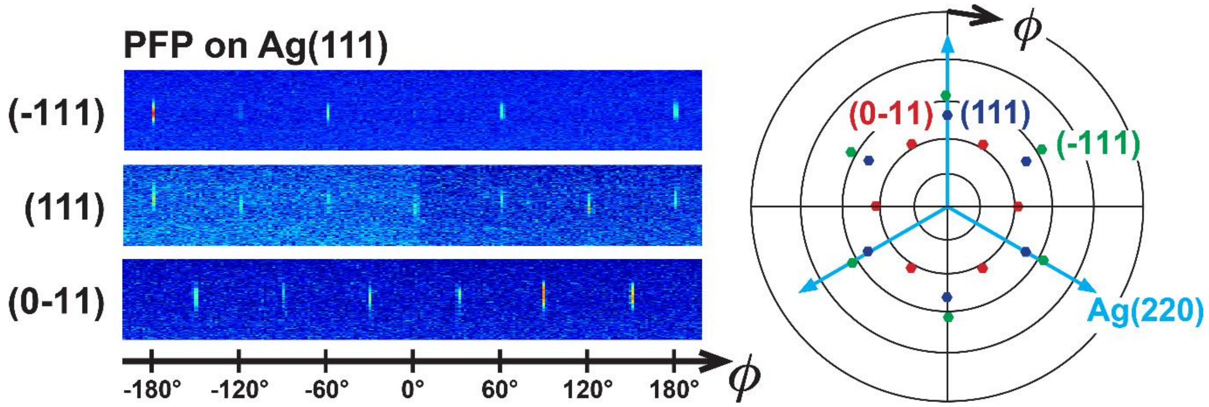

Figure S7: Left: azimuthal scans (rotation of the sample around the surface normal axis by the angle $-180° < \phi < +180°$ at different inclination angles (χ) in pole figure geometry) of a nominally 30 nm thick PFP film on a Ag(111) single crystal. The azimuthal scans are performed at fixed 2θ values which are derived from the structure solution found for π-stacked PFP on HOPG [10]; the lattice spacings (d_{hkl}) corresponding to the reflections investigated in the pole figures are d_{-111} = 5.686 Å, d_{111} = 4.743 Å, and d_{0-11} = 4.587 Å in this structure. The angle ϕ = 0° is set to the angle found for the Ag(220) reflection of the single crystal substrate (not shown); the χ-angles with respect to the (001) texture plane of PFP on Ag(111), as deduced from specular X-ray diffraction, are: χ_{-111} = 46.511°, χ_{0-11} = 30.901°, and χ_{111} = 38.912°. The experimental ϕ-values match perfectly with the simulated data (right Figure), which allows deriving the epitaxial relationship between adsorbate and substrate to: $(001)_{PFP}||(111)_{Ag}$ and $[1\text{-}10]_{PFP}||[1\text{-}10]_{Ag}$. From our pole figure analysis the growth of PFP in the π-stacked polymorph on Ag(111) becomes evident. Experiments have been performed at the German Electron Synchrotron (DESY, Hasylab beamline W1), the wavelength was set to λ = 1.2033 Å.

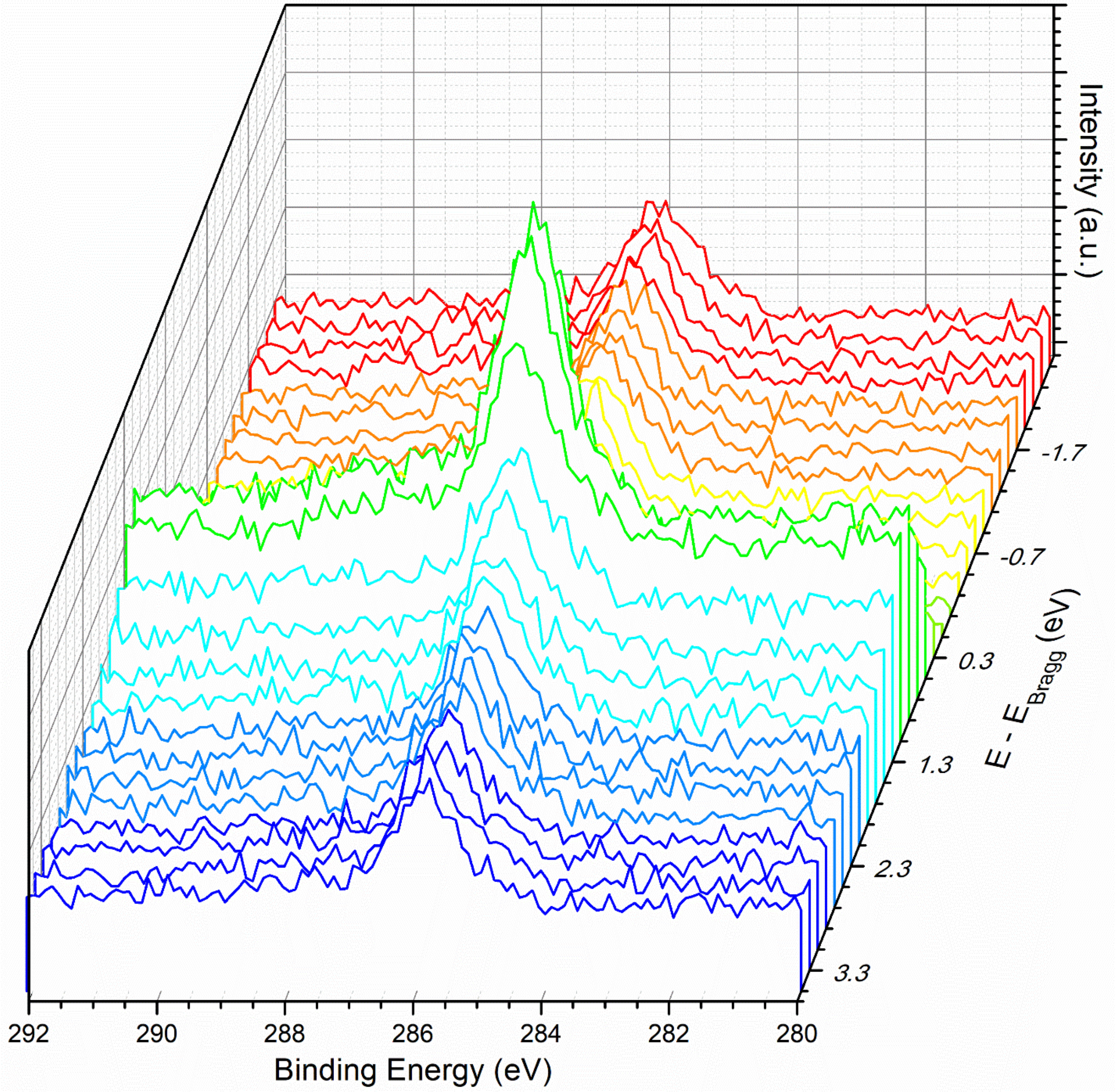

Figure S8: A set of XSW measurements of F4PEN on Ag(111) single crystal, C 1s core-level spectra, which generates the C 1s Y_P curve of high coverage in **Fig. S1**. The X-axis is binding energy, the same as in the XPS spectra; Y-axis the intensity, usually of arbitrary unit; Z-axis the difference between the photon energy (E) and the Bragg condition (E_{Bragg}), as each curve is recorded around the Bragg condition.

References

1. Zegenhagen, J. *Surf. Sci. Rep.* **1993,** *18,* 202–271. doi:10.1016/0167-5729(93)90025-K
2. Zegenhagen, J. *Surf. Sci.* **2004,** *554,* 77–79. doi:10.1016/j.susc.2003.12.057
3. Gerlach, A.; Bürker, C.; Hosokai, T.; Schreiber, F. X-Ray Standing Waves and Surfaces X-Ray Scattering Studies of Molecule–Metal Interfaces. In *The Molecule–Metal Interface*; Koch, N.; Ueno, N.; Wee, A. T. S., Eds.; Wiley-VCH, 2013; pp 153–172. doi:10.1002/9783527653171.ch6
4. Scilab, Free and open source software for numerical analysis, in: S. Enterprises (Ed.), https://www.scilab.org/, 2018.
5. Gerlach, A.; Schreiber, F.; Sellner, S.; Dosch, H.; Vartanyants, I. A.; Cowie, B. C. C.; Lee, T.-L.; Zegenhagen, J. *Phys. Rev. B* **2005,** *71,* 205425. doi:10.1103/PhysRevB.71.239902
6. Woodruff, D. P. *Rep. Prog. Phys.* **2005,** *68,* 743–798. doi:10.1088/0034-4885/68/4/R01
7. Duhm, S.; Burker, C.; Niederhausen, J.; Salzmann, I.; Hosokai, T.; Duvernay, J.; Kera, S.; Schreiber, F.; Koch, N.; Ueno, N.; Gerlach, A. *ACS Appl. Mater. Interfaces* **2013,** *5,* 9377–9381. doi:10.1021/am402778u
8. Duhm, S.; Hosoumi, S.; Salzmann, I.; Gerlach, A.; Oehzelt, M.; Wedl, B.; Lee, T.-L.; Schreiber, F.; Koch, N.; Ueno, N.; Kera, S. *Phys. Rev. B* **2010,** *81,* 045418. doi:10.1103/PhysRevB.81.045418
9. Gerlach, A.; Hosokai, T.; Duhm, S.; Kera, S.; Hofmann, O. T.; Zojer, E.; Zegenhagen, J.; Schreiber, F. *Phys. Rev. Lett.* **2011,** *106,* 156102. doi:10.1103/PhysRevLett.106.156102
10. Salzmann, I.; Moser, A.; Oehzelt, M.; Breuer, T.; Feng, X.; Juang, Z.; Nabok, D.; Della Valle, R. G.; Duhm, S.; Heimel, G.; Brillante, A.; Venuti, E.; Bilotti, I.; Christodoulou, C.; Frisch, J.; Puschnig, P.; Draxl, C.; Witte, G.; Müllen, K.; Koch, N. *ACS Nano* **2012,** *6,* 10874–10883. doi:10.1021/nn3042607
